# Supplementary material for: Validating agent-based simulation model of hospital-associated Clostridioides difficile infection using primary hospital data
Source: PLoS One. 2023 Apr 21;18(4):e0284611. doi: 10.1371/journal.pone.0284611 (PMC10120937; doi:10.1371/journal.pone.0284611)
Supplement: S1 Appendix — (DOCX) [file pone.0284611.s001.docx]

**SUPPLEMENTARY MATERIAL**

**Supplement section A. H-ABM Logic and Assumptions**

H-ABM draws upon previously published ABMs of the spread of hospital-associated *C. difficile* in a generic hospital model, particularly Codella (2015), and Barker (2018)^1,2^. These models replicate the events and conditions related to the spread of *C. difficile* in a small, community-hospital type setting. To create H-ABM, we expanded this generic model considerably, but maintained several of the modeling logic and assumptions from the generic model.

*C. difficile propagation*

In both H-ABM and the generic model, a patient develops CDI via exposure to *C. difficile* spores. Once a patient has been exposed, the progression of CDI follows from the DTMC. Patients may be exposed to *C. difficile* spores through interactions with contaminated agents (i.e., doctors, nurses, visitors, and other patients), or environments. The H-ABM and generic model simulates each of these types of interactions explicitly, and conducts Bernoulli “coin-flip” trials to assess the success of exposure if one of the interacting agents/environment is contaminated with *C. diff* spores. Probability of exposure increases with length of interaction and the rate of physical contact. For example, we model the probability that a nurse whose hands are currently contaminated with *C. difficile* spores will expose a patient to *C. difficile* by the following equation.

$$p_{\mathrm{np}}= \left( \left( 1 - \exp\left( -\lambda_{\mathrm{np}}* \mathrm{LO}S_{n} \right) \right) * \alpha_{\mathrm{np}} \right)$$

Where:

$$p_{np}=\text{Probability for nurse-to-patient exposure (without infection control intervention)}\text{}$$

$$\lambda_{np}=\text{Rate of contact between nurses and patients}\text{}$$

$$LOS_{n}=\text{Length of stay of nurse in patient room}$$

$$\alpha_{np}=\text{Transfer efficiency between one individuals hands to another's}$$

H-ABM uses *p_np_* and the probability of success for the relevant infection control intervention to determine if *C. difficile* is propagated. To expand the example above, the model then follows the procedure illustrated below, where *PHH* represents the probability of a successful patient hand hygiene event (i.e., the product of patient hand hygiene compliance and effectiveness):

1. Generate two random numbers on interval [0,1]: designate as *r_1,_ r_2_*
2. Assess if *r_1_* ≤ *p_np_* AND *r_2_* ≤ *PHH*
3. If TRUE, exposure successful; else, exposure unsuccessful.

If exposure is successful, the patient disease state is updated to the “Exposed” state illustrated in Figure 1, then updated every six hours in-simulation according the DTMC transition probability matrix. Similar computations are used to assess contamination in each agent/environment interaction.

Interactions with the environment are not explicitly modeled and are assumed to occur with infinite contact rate. Therefore, probability of transmission of *C. difficile* spores via environment is dependent only on duration of stay in a particular environment. Appendix Figure A1-A3 illustrate the logic used to update each type of agent in the H-ABM and the generic model, including relevant interactions with other agents.

*C. difficile infection transition probability matrix*

The H-ABM and the generic model use a time step of 5 minutes in-simulation. Every six hours in-simulation, the disease state of each patient is updated according to a discrete-time Markov Chain (DTMC). The DTMC transition probability matrix used in the H-ABM governs the transitions illustrated in Figure 1 of the manuscript and is one of 10 candidate matrices developed in Codella, et al. These 10 matrices were produced by calibrating the generic hospital model to historical CDI rate data and selecting the best performing matrices, as evaluated by mean percentage error across multiple outcomes of interest [1]. We emphasize that all transitions shown with solid arrows Figure 1 are independent of the hospital state and the infection control interventions in place. The two exceptions are detailed below:

1. Non-susceptible to susceptible: this transition is only possible if a patient begins a course of antibiotics during their stay in the hospital. Therefore, patient agents that start a course of antibiotics are updated to the ‘susceptible’ state when their antibiotics start.
2. Susceptible to exposed: this transition is only possible if a patient is exposed to *C.difficile* spores through interaction with another agent or the environment. The prevalence of *C. difficile* in the hospital, which is affected by infection control interventions, is the primary driver of this transitions. Patients transition to ‘exposed’ as soon as an exposure occurs in the simulation.

*Supplement Figure A1: Patient agent logic (simplified)*

*
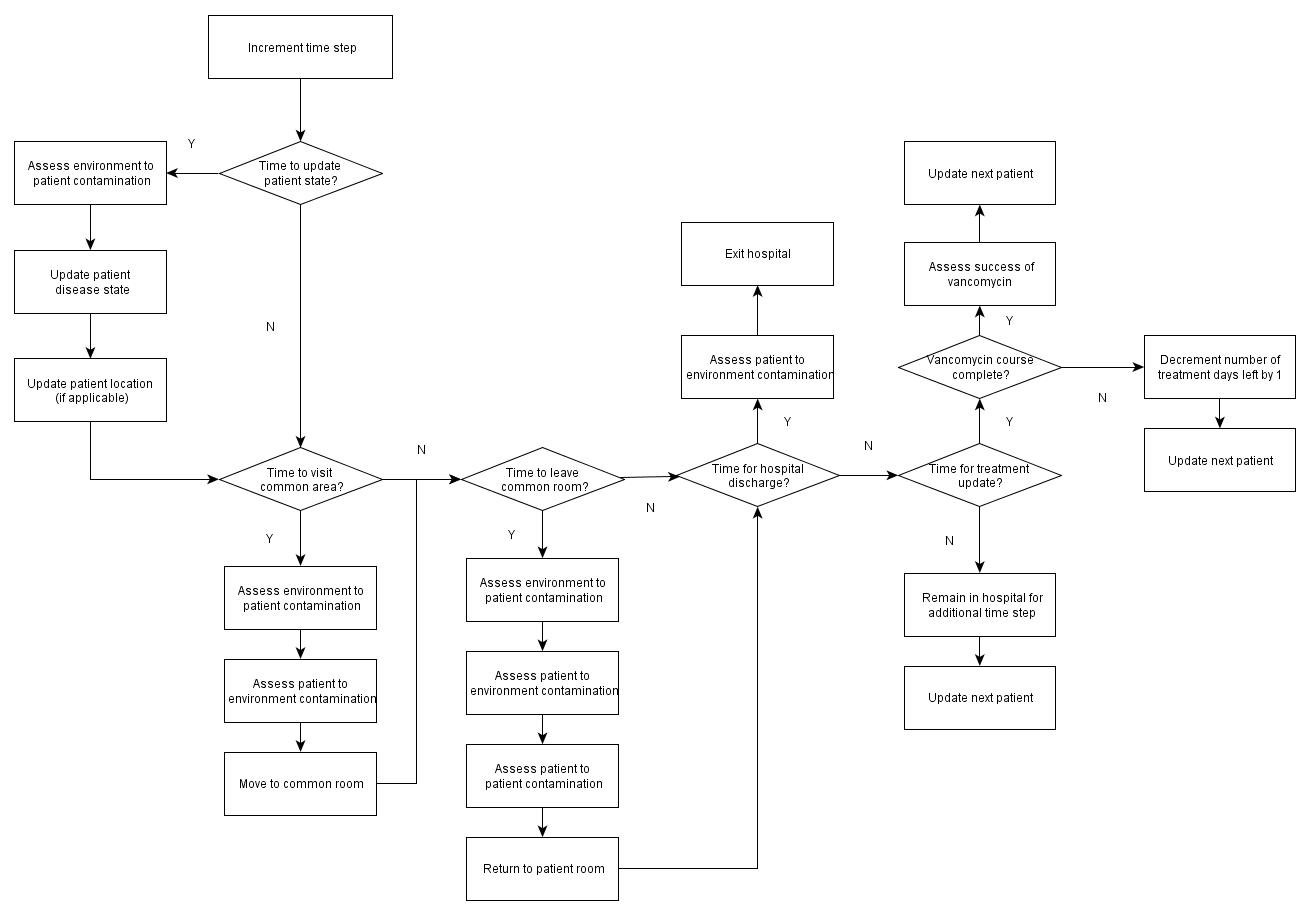
*

*Supplement Figure A2: HCW (nurses and doctors) agent logic (simplified)*

*
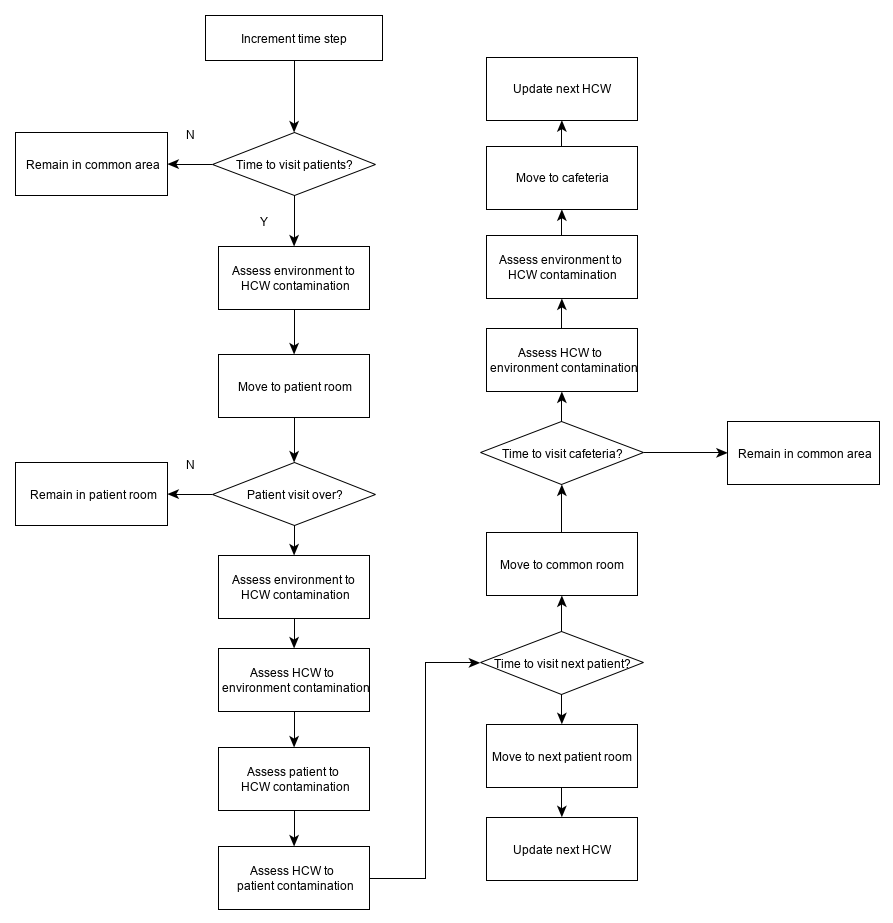
*

N

N

Y

Y

*Supplement Figure A3: Hospital visitor agent logic (simplified)*

**
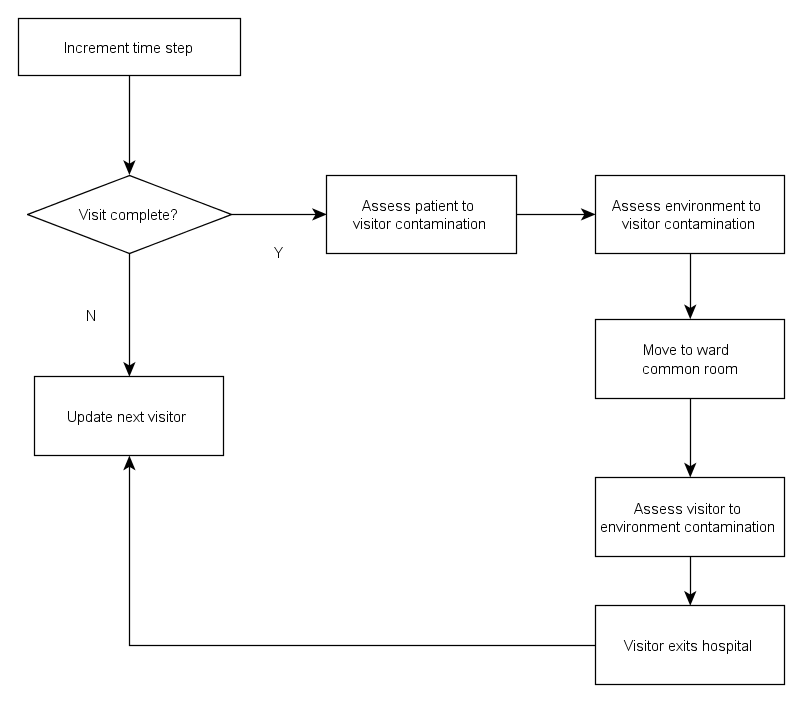
**

**Supplement section B.** **Description of primary hospital data**

The data used to build the H-ABM and estimate infection control intervention parameters was obtained from two sources: a database of patient electronic health records, and the records of the target hospital’s infection control program.

From the database of target hospital patient electronic health records, we obtained summary statistics across 157,507 patient admissions from the years 2013-2018 (inclusive). We only considered records from patients over the age of 18 who were admitted as inpatients. From this dataset, we obtained the following counts, aggregated by each year and by each ward in the target hospital:

- Total number of admissions,
- Total number of patients over the age of 65 years,
- Total number of patients given a high-risk antibiotic (clindamycin, ceftriaxone, carbapenems, or fluoroquinolones) during their stay,
- Average length of stay,
- Total number of patients with an active high-risk antibiotic (clindamycin, ceftriaxone, carbapenems, or fluoroquinolones) prescription on admission.

We define an antibiotic as ‘given’ if there is a medication administration record (MAR) containing one of the following codes:

| MAR Code | Description |
| --- | --- |
| 1 | Given |
| 6 | New Bag |
| 7 | Restarted |
| 9 | Rate Change |
| 12 | Bolus |
| 13 | Push |
| 100 | Due |
| 104 | Bolus from Bag/Injection |
| 106 | Restarted IV |
| 113 | Continue from OR/PACU/Procedure |
| 150 | Given Early |
| 151 | Given Late |
| 157 | Downtime New Bag/Injection |
| 158 | Downtime Given |
| 159 | Documented in Another Care Area |
| 166 | Change Bag/Injection |
| 175 | Bolus |
| 176 | Bolus from Bag/Injection |

These fields were delivered by the target hospital’s translational research data team. No patient-identifying data was delivered or used in this study.

The second source of data was the target hospital’s infection control program, which records counts of the number of observed hand-hygiene events across the target hospital every month. The infection control program provided the following data, aggregated by the year (for 2013-2018, inclusive) and by each ward in the target hospital:

- Number of Hospital-associated CDIs per 10,000 patient days
- Number of hand hygiene events recorded, performed by doctors,
- Number of hand hygiene events recorded, performed by nurses,
- Number of hand hygiene events recorded using alcohol-based hand sanitizer rub, performed by doctors,
- Number of hand hygiene events recorded using alcohol-based hand sanitizer rub, performed by nurses,
- Number of hand hygiene events recorded using soap-and-water, performed by doctors,
- Number of hand hygiene events recorded using soap-and-water, performed by nurses.

From these counts, we obtained probabilities of doctor/nurse compliance with hand hygiene practices for each year. We also obtained probabilities for use of alcohol-based hand sanitizer rubs vs soap-and-water for each hand hygiene event, for both nurses and doctors. As this data is proprietary, we aggregate the estimates of nurse and doctor hand hygiene compliance as “health care worker” hand hygiene compliance for presentation in this study. However, the H-ABM uses separate doctor and nurse hand hygiene compliance and effectiveness parameters.

**Supplement section C. Further changes in H-ABM**

Barker, et al and Codella, et al previously published two generic models of hospital-associated CDI in adults [1,2]. To create H-ABM we drew upon several of the assumptions inherent in previous models and introduced new structures. The first major difference between the Barker, et al model and the H-ABM is the programming language used to create each model. The Barker, et al model was built in NetLogo, but the H-ABM was created in Java [3]. Java is a very flexible programming language and allows us to run replications of the H-ABM relatively quickly. A detailed description of the NetLogo to Java translation is included in Scaria, et al [4].

Another of these changes is the update to modeled hospital layout. Barker, et al models a hospital comprising 10 wards, each with 20 patient rooms. To mirror the layout of the target hospital, H-ABM comprises 18 wards of heterogeneous size, ranging from 8 to 41 patient rooms. To obtain an accurate representation of the target hospital, a research group member performed a walk-through of the hospital and recorded the number of patient rooms and common areas. The generic models assume that each ward has exactly one doctor’s common room, one nurse’s common room, and one visitor’s/patient’s common room. H-ABM allows for multiple common rooms of any type in each ward, and includes additional common areas shared by nurses and doctors, where applicable. Rooms for patients with negligible CDI risk (e.g., psychiatry, sleep studies), those that primarily serve outpatients, or that are sporadically occupied (e.g., rooms reserved for incarcerated patients) were omitted from the model. Healthcare worker common areas typically visited only at the beginning or end of a working shift, such as locker rooms, were omitted from the model. Hallways and other areas between patient rooms were also omitted from the model because of the short agent length of stay in these areas.

In the H-ABM, patients are admitted to the hospital wards according to a probability distribution created from historical target hospital admissions data. We assume that patients staying in a ward are admitted to the hospital for needs corresponding to the ward’s specialty and use these specialties to help assess the CDI risk of those patients. Depending on the demographics, antibiotic use, and risk associated with each ward’s specialty, patients are assigned as susceptible, colonized, infected, or non-susceptible upon admission. Length of stay varies depending on the patient’s home ward. The H-ABM also adjusts patient susceptibility depending on whether a patient is taking an antibiotic associated with risk of CDI on the second day of their hospital stay.

The H-ABM also includes enhanced intervention logic for particular wards. The section of patient rooms for palliative care within a single ward requires healthcare workers to perform hand hygiene before entry. Also, following the infection control improvements made in 2016, bone marrow transplant patients were screened for CDI upon admission to the hospital; this translates to testing all patient agents assigned to a specific ward in the H-ABM at admission. To account for variation in healthcare worker hand hygiene across wards, we used by-ward historical rates of hand hygiene to estimate by-ward hand hygiene multipliers to modify hand hygiene parameters.

Because primary data pertaining to environmental cleaning compliance was not available, we needed to estimate parameters with a combination of estimates from literature and hospital data of other interventions. To account for the uncertainty in these estimates, we split the parameters of terminal and daily cleaning compliance into three parameters describing the minimum, maximum, and average compliances. For each environmental cleaning event, we sampled the probability of success (i.e., a cleaning event occurs and successfully removes *C. difficile* spores) from a symmetric triangular distribution parametrized by the minimum, maximum, and mean compliance estimates.

Two of the most important risk factors for developing CDI are general susceptibility (usually caused by advanced age, immunosuppression, etc.), and antibiotic use. In order to incorporate how the antibiotic prescribing practices and the susceptible population change over time, we used primary hospital data to obtain estimates for the proportion of patients susceptible to CDI and the proportion of patients who are administered antibiotics during their stay. These proportions varied over the years, with antibiotic use showing a marked decline in the target hospital. Therefore, to incorporate these time-dependent changes into the ABM, we developed scalar modifiers for each year that would be multiplied with the vectors of susceptibility and antibiotic use in Supplement Table C2 to develop yearly estimates of antibiotic use and susceptible population. These modifiers are included in Supplement Tables C3 and C4.

*Supplement Table C1: Input parameters unchanged from Barker, et al* [2] *(Adapted from Scaria, et al* [4]*)*

| Admission Parameters | | |
| --- | --- | --- |
| **Parameter** | **Mean value** | **Source** |
| Proportion of asymptomatic colonized patients | 6.10% | [5–15] |
| Proportion of patients with CDI | 0.29% | [5,12,16,17] |
| Health care worker behavior and distribution | | |
| **Parameter** | **Value** | **Source** |
| Patient-nurse contact probability [rate] | 0.358 (for 5 minutes) [10.53 contacts/ minute] | [18] |
| Patient-doctor contact probability [rate] | 0.688 (for 5 minutes) [9.25 contacts/ minute] | [18] |
| Number of nurses per 20 patient rooms | 4 | [18–21] |
| Number of doctors per 20 patient rooms | 2 | [18,22] |
| Average nurse service time | 4.7 minutes | [18,23–25] |
| Average doctor service time | 10.8 minutes | [18,23–25] |
| Average number of nurse visits per 6 hours | 5 | [18,25–27] |
| Average number of doctor visits per 6 hours | 1 | [18,25–27] |
| Visitor behavior | | |
| **Parameter** | **Mean value** | **Source** |
| Probability of receiving visitors (per day) | 0.5 | [28,29] |
| Number of visitors per visit | 2 | [29,30] |
| Visitor length of stay | 15 minutes | [18,29–31] |
| Visitor-environment contact probability [rate] | 0.932 (for 15 minutes) [0.179 contacts/ minute] | [18] |

*Supplement Table C2: Ward-specific input parameters used in H-ABM for the year 2013*

| Ward Number | Susceptible, % | Non-Susceptible, % | Average length of stay (days) | Antibiotic use (baseline), % | Number of patient rooms | Number of Nurses | Number of Doctors |
| --- | --- | --- | --- | --- | --- | --- | --- |
| 1 | 93.65% | 0.00% | 5.32 | 42.10% | 24 | 5 | 3 |
| 2 | 29.09% | 64.56% | 7.51 | 21.05% | 8 | 3 | 2 |
| 3 | 43.04% | 50.61% | 1.97 | 23.34% | 28 | 6 | 3 |
| 4 | 38.55% | 55.10% | 4.68 | 17.73% | 26 | 6 | 3 |
| 5 | 42.84% | 50.81% | 4.72 | 17.10% | 26 | 6 | 3 |
| 6 | 43.34% | 50.31% | 3.94 | 19.99% | 27 | 6 | 3 |
| 7 | 39.58% | 54.07% | 7.44 | 19.39% | 18 | 4 | 2 |
| 8 | 74.98% | 18.67% | 5.36 | 40.87% | 28 | 6 | 3 |
| 9 | 56.15% | 37.50% | 3.06 | 10.22% | 21 | 5 | 3 |
| 10 | 93.65% | 0.00% | 6.43 | 44.21% | 28 | 6 | 3 |
| 11 | 58.02% | 35.63% | 4.82 | 23.77% | 15 | 3 | 2 |
| 12 | 53.20% | 40.45% | 7.05 | 30.45% | 20 | 4 | 2 |
| 13 | 42.35% | 51.30% | 4.32 | 33.36% | 28 | 6 | 3 |
| 14 | 41.39% | 52.26% | 6.34 | 34.21% | 28 | 6 | 3 |
| 15 | 0.00% | 93.65% | 1.43 | 0.83% | 11 | 3 | 2 |
| 16 | 51.77% | 41.88% | 4.84 | 37.32% | 18 | 4 | 2 |
| 17 | 93.65% | 0.00% | 10.74 | 63.47% | 41 | 9 | 5 |
| 18 | 93.65% | 0.00% | 9.45 | 87.08% | 31 | 7 | 4 |

*Supplement Table C3: Yearly scalar modifier for average antibiotic use*

| Year | Average antibiotic use modifier (relative to 2013) |
| --- | --- |
| 2013 | 1.00 |
| 2014 | 0.91 |
| 2015 | 0.84 |
| 2016 | 0.78 |
| 2017 | 0.65 |
| 2018 | 0.66 |

*Supplement Table C4: Yearly scalar modifier for susceptible population*

| Year | Susceptible population modifier (relative to 2013) |
| --- | --- |
| 2013 | 1.00 |
| 2014 | 0.97 |
| 2015 | 0.98 |
| 2016 | 0.94 |
| 2017 | 0.98 |
| 2018 | 0.92 |

**Supplement section D. CDI testing algorithm and infection control interventions**

In response to high HA-CDI rates in 2013 and 2014, the target hospital initiated several new infection control interventions beginning in 2015 and 2016. In particular, the hospital implemented a novel CDI testing algorithm in 2015 to reduce the number of false classifications of HA-CDIs [32]. The Centers for Disease Control and Prevention (CDC) guidelines classify all CDI cases tested positive within three days of admission as CA-CDI and any cases diagnosed after this time as HA-CDI. The new CDI testing algorithm promotes increased CDI testing during the first 48 hours of a patient’s stay. The algorithm also encourages clinicians to consider alternate causes of gastrointestinal symptoms prior to testing after 48 hours of a stay and limits the number of diagnostic tests to one test every seven days.

The CDI Testing Algorithm was implemented in the target hospital towards the end of 2015. The testing algorithm was proposed and created after an extensive chart review found several patients had either been inappropriately tested for CDI (e.g., had gastrointestinal symptoms, but were taking laxatives). Inappropriately tested patients not only strain the hospital’s laboratory resources and receive unnecessary antibiotic treatment, but could also lead to asymptomatic colonizations (which are not typically reported by hospitals) being classified as HA-CDIs [32,33]. The chart review also revealed that several patients had gastrointestinal symptoms at admission, but were tested late into their stay. These patients tested late might have been exposed to *C. difficile* in the community and might have been classified as CA-CDIs instead of HA-CDIs. Late testing may also delay important treatment and lead to increased spread of *C. difficile* spores. These inappropriate and/or late tests caused the rate of HA-CDI to be inaccurately high. Since large academic hospitals can be penalized by the Centers for Medicare & Medicaid Services for high HA-CDI rates, reporting accurate HA-CDI rates is of great importance [34].

To represent the CDI testing algorithm in our model (Supplement Figure D1), we needed to estimate the number of patients tested in the hospital whose gastrointestinal symptoms had non-CDI causes (such as laxative or stool softener use). We assumed that community and hospital prevalence of non-CDI gastrointestinal symptoms did not change during 2013-2018. We also assumed that count of CDI tests in the years 2013-2015 includes patients that were inappropriately tested; similarly, we assumed that the count of CDI tests for the years 2016-2018 does not include inappropriately tested patients. We estimated the proportion of these inappropriately tested patients using the numbers of CDI tests administered within 48 hours of a patient’s stay before and after the testing algorithm was implemented in late 2015. Similarly, we estimated the proportion of patients with CDI that might have non-CDI related causes for their gastrointestinal symptoms using the change in the number of tests administered after 48 hours of a patient’s stay before and after algorithm implementation.

*Supplement Figure D1: CDI testing algorithm logic, as implemented in H-ABM*

*
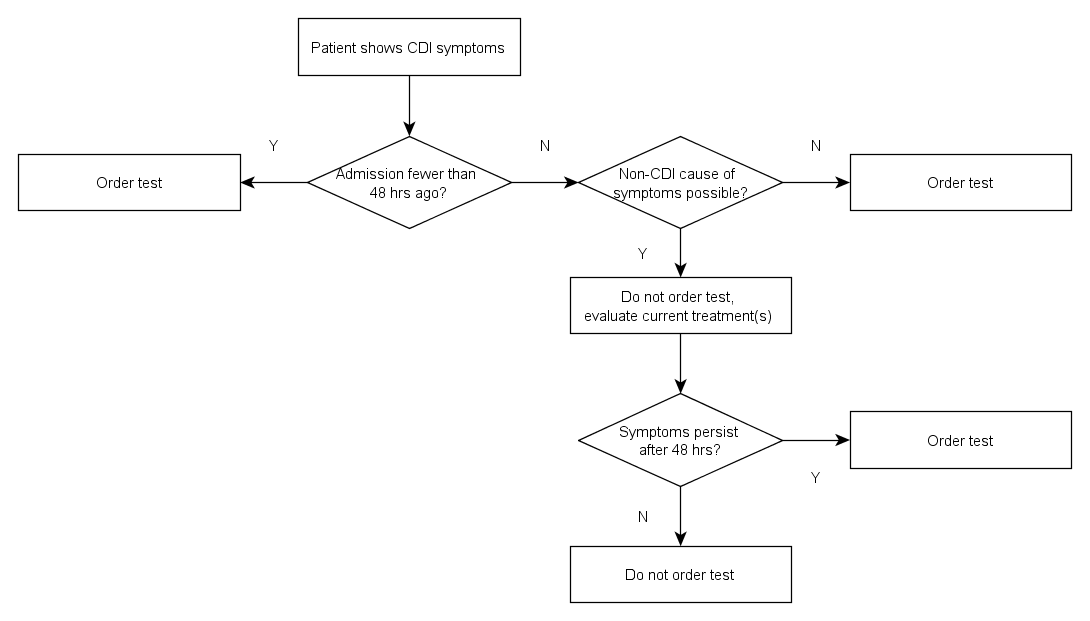
*

Additional measures implemented in 2015 including a fluoroquinolone restriction in the trauma and solid organ transplant wards; screening asymptomatic bone marrow transplant patients upon admission; increased use of supplemental ultraviolet disinfection following all CDI discharges and transfers; deployment of ultraviolet disinfection in some non-CDI rooms. Moreover, in 2016, the target hospital devoted additional resources to improving infection control intervention compliance. For example, hospital infection control started a program of direct observations for contact precautions and hand hygiene to improve adherence.

**Supplement section E. Parameter estimation**

Intervention parameters in H-ABM are estimated through a combination of primary hospital data and literature. Since 2012, the target hospital has kept a record of hand hygiene compliance for nurses and doctors. This record differentiates between alcohol-based hand rub (ABHR) and soap & water hand hygiene events. This primary data was self-reported and likely overrepresents the compliance with hand hygiene protocols. To mitigate the effects of overrepresentation, we assumed the hospital hand hygiene data represents the rate of hand hygiene compliance of nurses and doctors when interacting with a known CDI patient.

To obtain estimates for all other intervention parameters, we needed to scale estimates drawn from literature using hospital hand hygiene data. The equation used to obtain scaled parameter estimate *(i,j)* for year *i* and intervention *j* is shown below.

$$Scaled estimate(i,j) = \frac{{Average}_{w}(Obs nurse HH (i), Obs doctor HH(i))\times Base(i,j)}{{Average}_{w}(Base nurse HH (i),Base doctor HH (i))}$$

Obs = observed, HH = hand hygiene

Where,

- ${Average}_{w}(Obs nurse HH (i), Obs doctor HH(i))$ is the weighted average of observed healthcare worker hand hygiene compliance derived from hospital hand hygiene data,
- ${Average}_{w}(Base nurse HH (i),Base doctor HH)$ represents the weighted average healthcare worker hand hygiene compliance when interacting with known CDI patients, as estimated from literature, and
- $Base(i,j)$ is an unscaled value of the parameter for year *i* and intervention *j*, as estimated from literature.

*Parameter estimation example*

We provide an example of our scaling methodology to obtain the estimate of patient standard hand hygiene compliance in the year 2013. Using data sourced from the target hospital infection control program, we estimate *Average_w_(Obs nurse HH(2013), Obs doctor HH(2013) =* 0.543. From literature, we estimate the average compliance of patients with hand hygiene protocols *Base(2013, patient hand hygiene (standard))* as approximately 0.22. To obtain this estimate, we consider multiple studies in literature quantifying patient hand hygiene compliance under conditions without significant investment from healthcare facilities. This selection reflects the condition of the target hospital in 2013, when there was diminished investment in hand hygiene improvement programs. We obtain *Average_w_(Base nurse HH(2013), Base doctor HH(2013)) =0.66* from literature. Similarly to the estimate for *Base(2013, patient hand hygiene (standard)),* we focus on literature describing healthcare worker hand hygiene compliance prior to any major hand hygiene improvement initiative. Following the equation provided this yields the scaled estimate for patient hand hygiene compliance in the year 2013 as 0.267, as included in Table 1.

**Supplement section F. Intervention Performance in H-ABM**

*Supplement Figure F1. Rate of HA-CDI per 10,000 patient days for each intervention vs baseline from H-ABM*


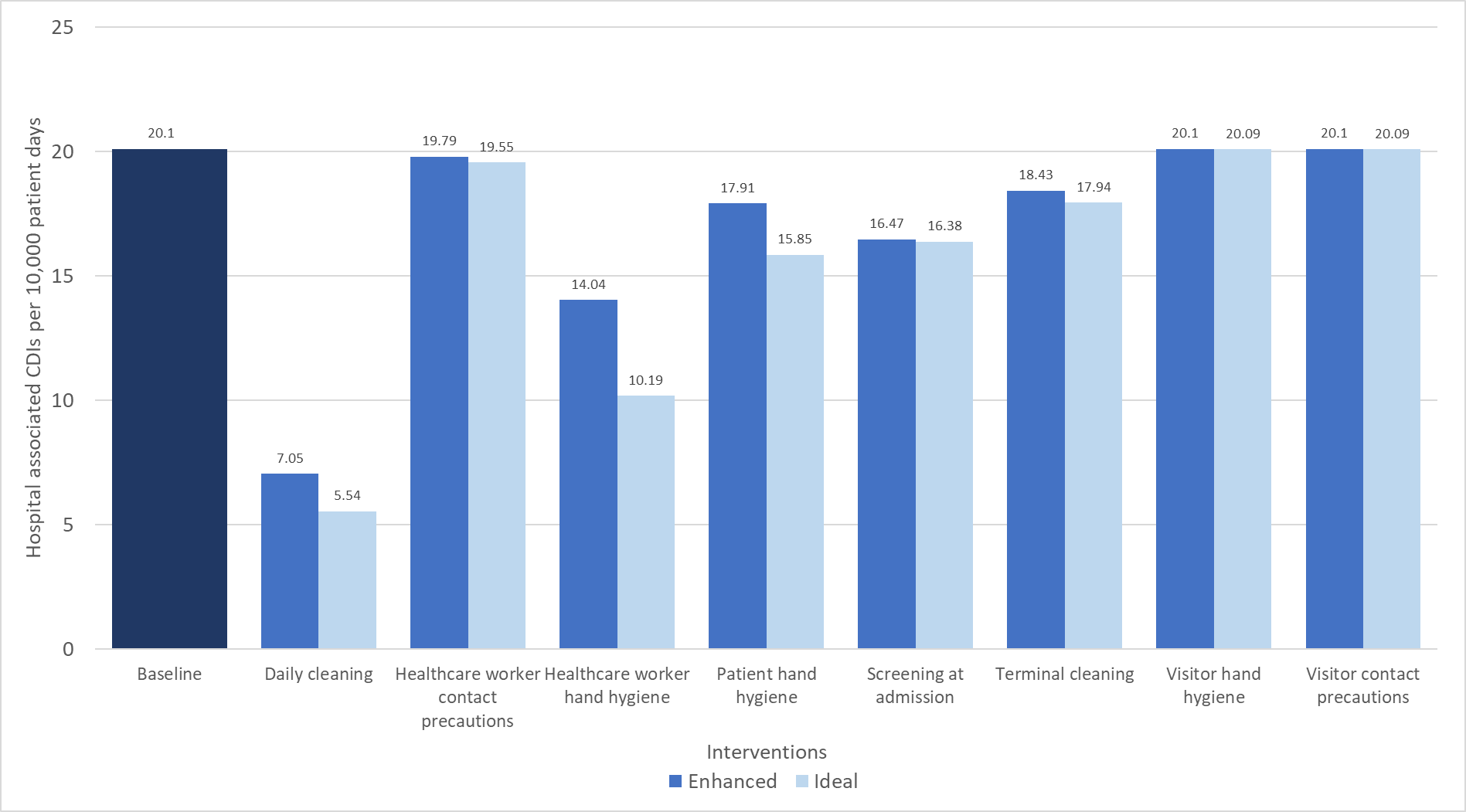


*Supplement Figure F2. Rate of asymptomatic colonization per 1,000 admissions for each intervention vs baseline from H-ABM*

**
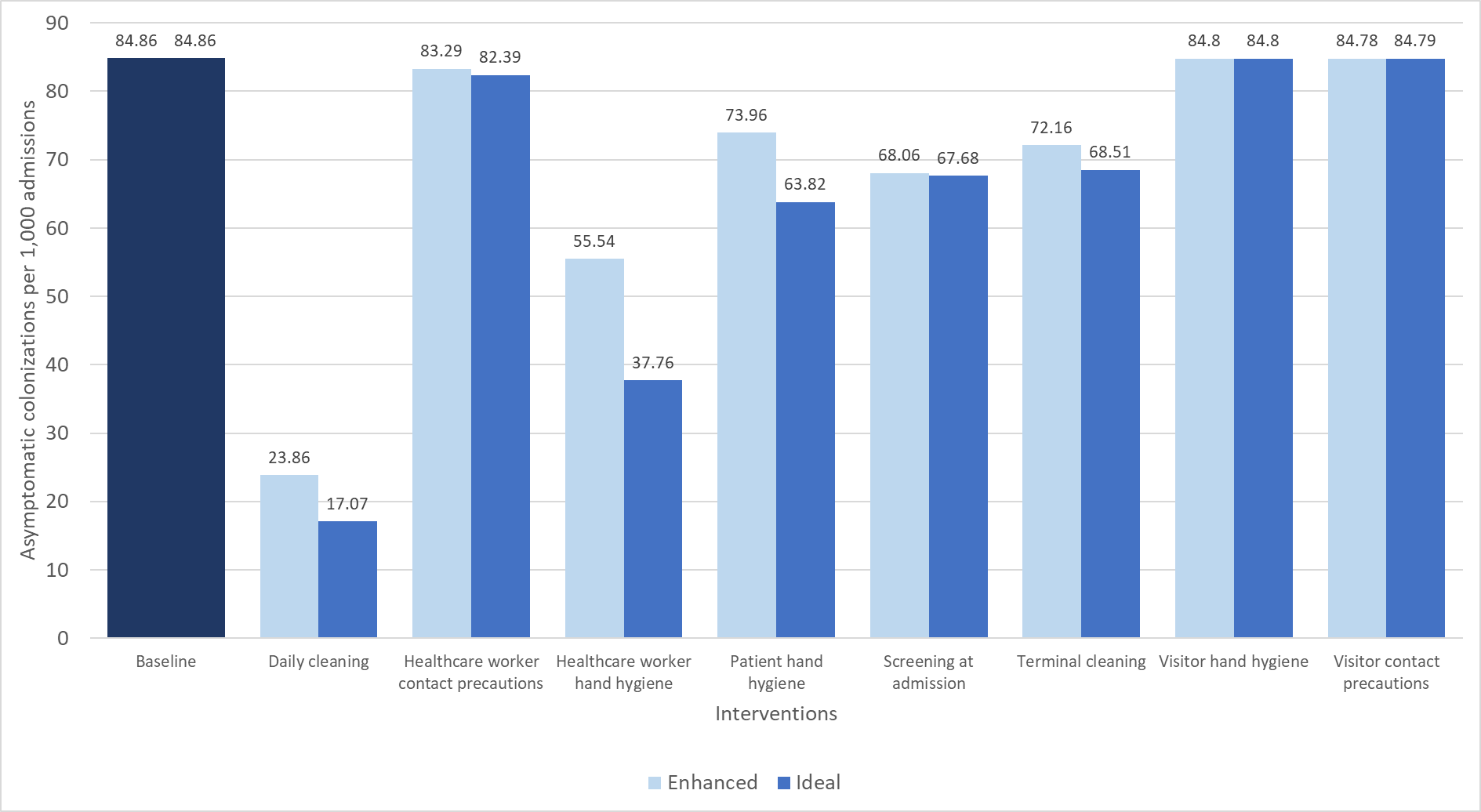
**

*Supplement Figure F3. Rate of HA-CDI per 10,000 patient days for each intervention vs baseline from the generic hospital model*

*
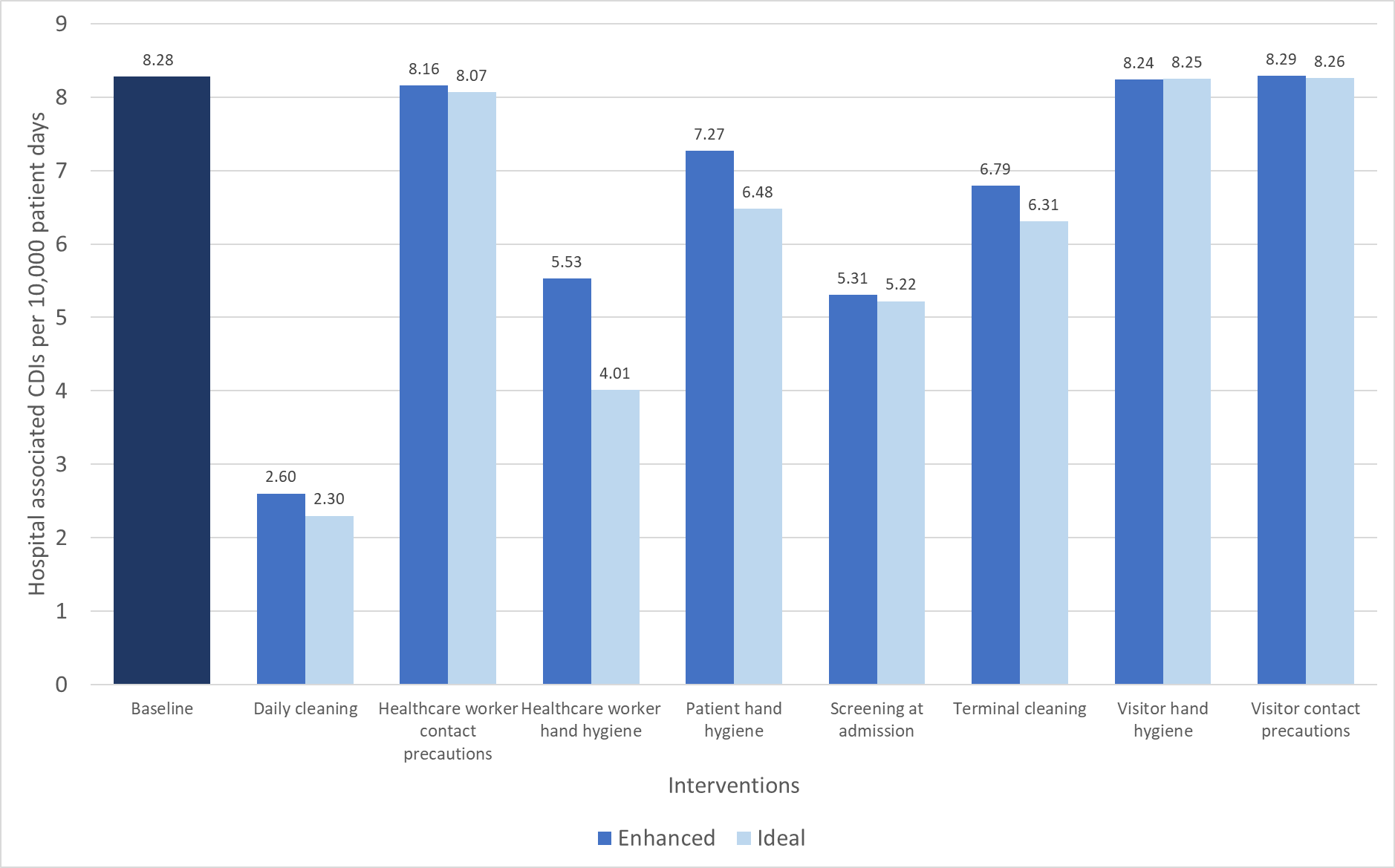
*

*Supplement Figure F4. Rate of HA-CDI per 10,000 patient days for each intervention vs baseline from the generic hospital model*

*
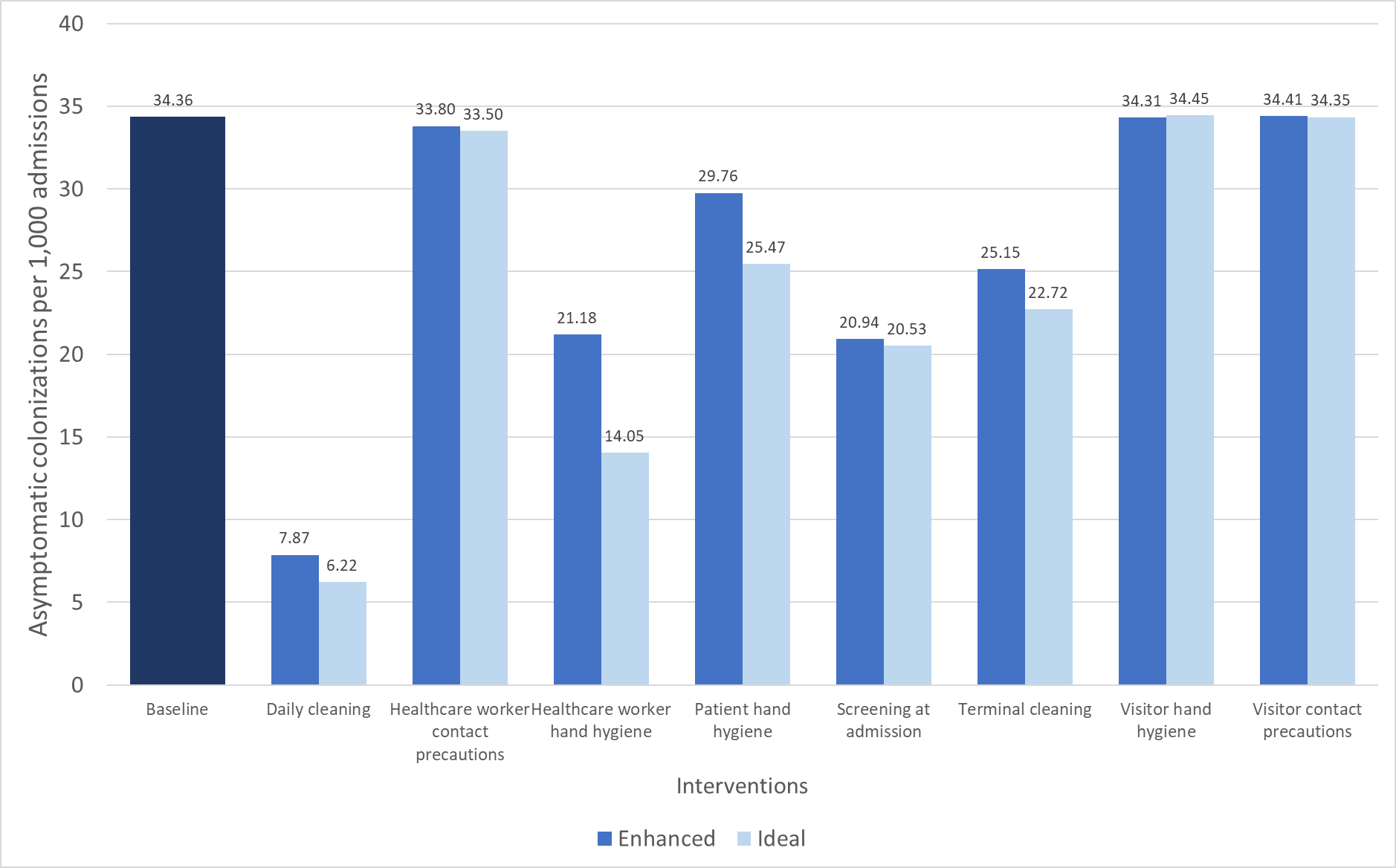
*

*Supplement Figure F5: Enhanced (a) and ideal (b) single intervention reduction in HA-CDI per 10,000 patient days, H-ABM vs generic model*

(a)

**
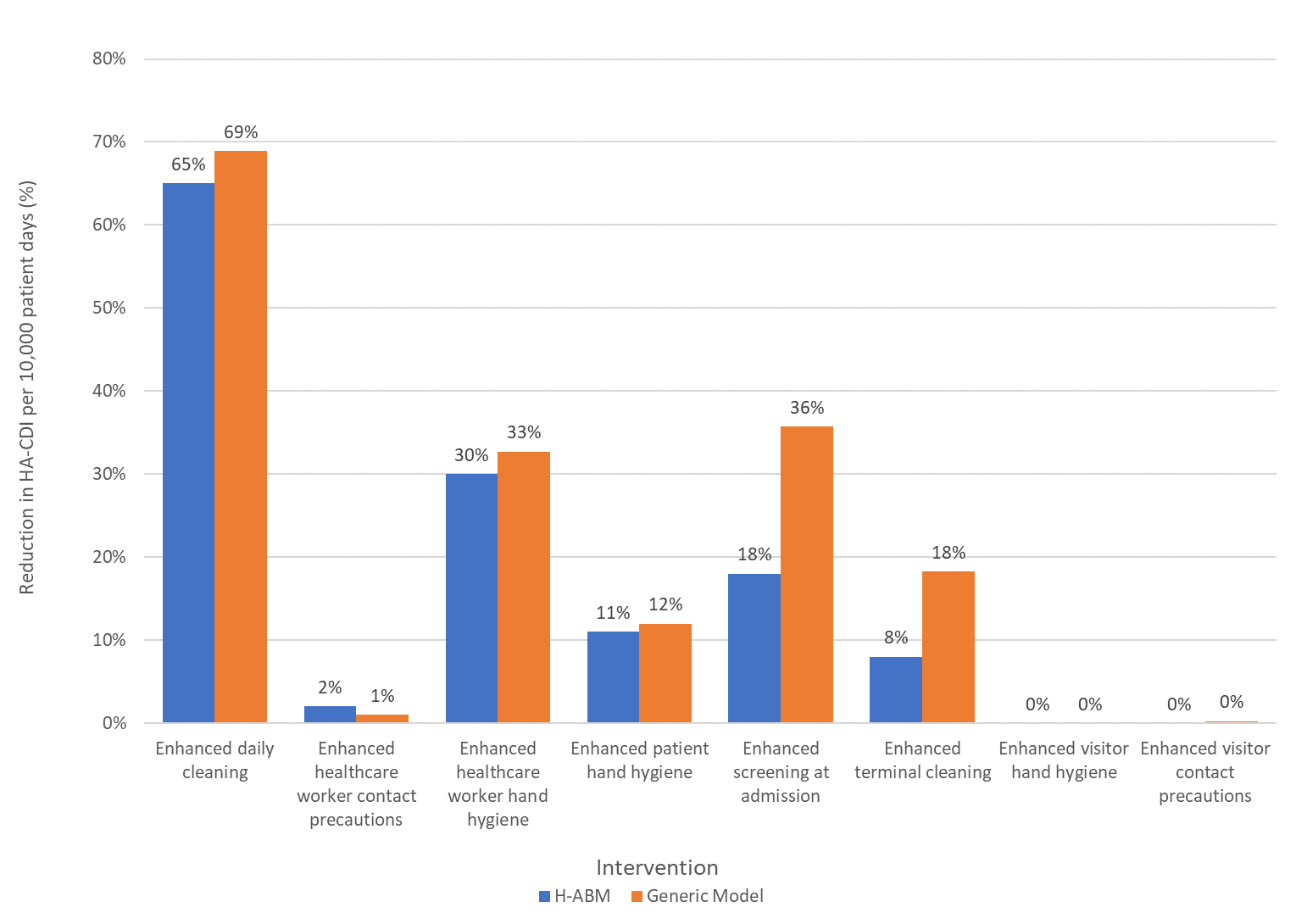
**

(b)


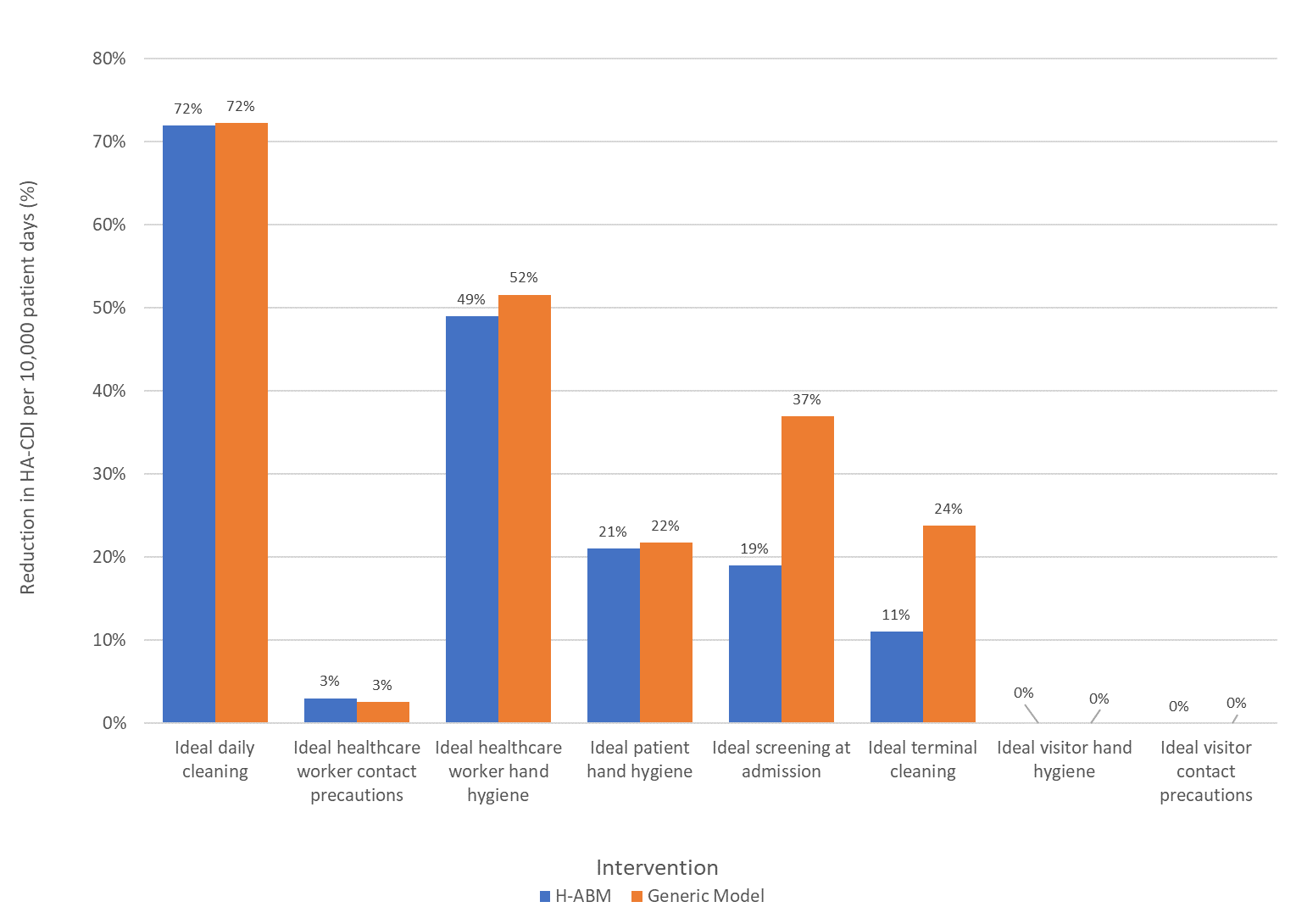


*Supplement Figure F6: Enhanced (a) and ideal (b) single intervention reduction in asymptomatic colonizations per 1,000 admissions, H-ABM vs generic model*

(a)


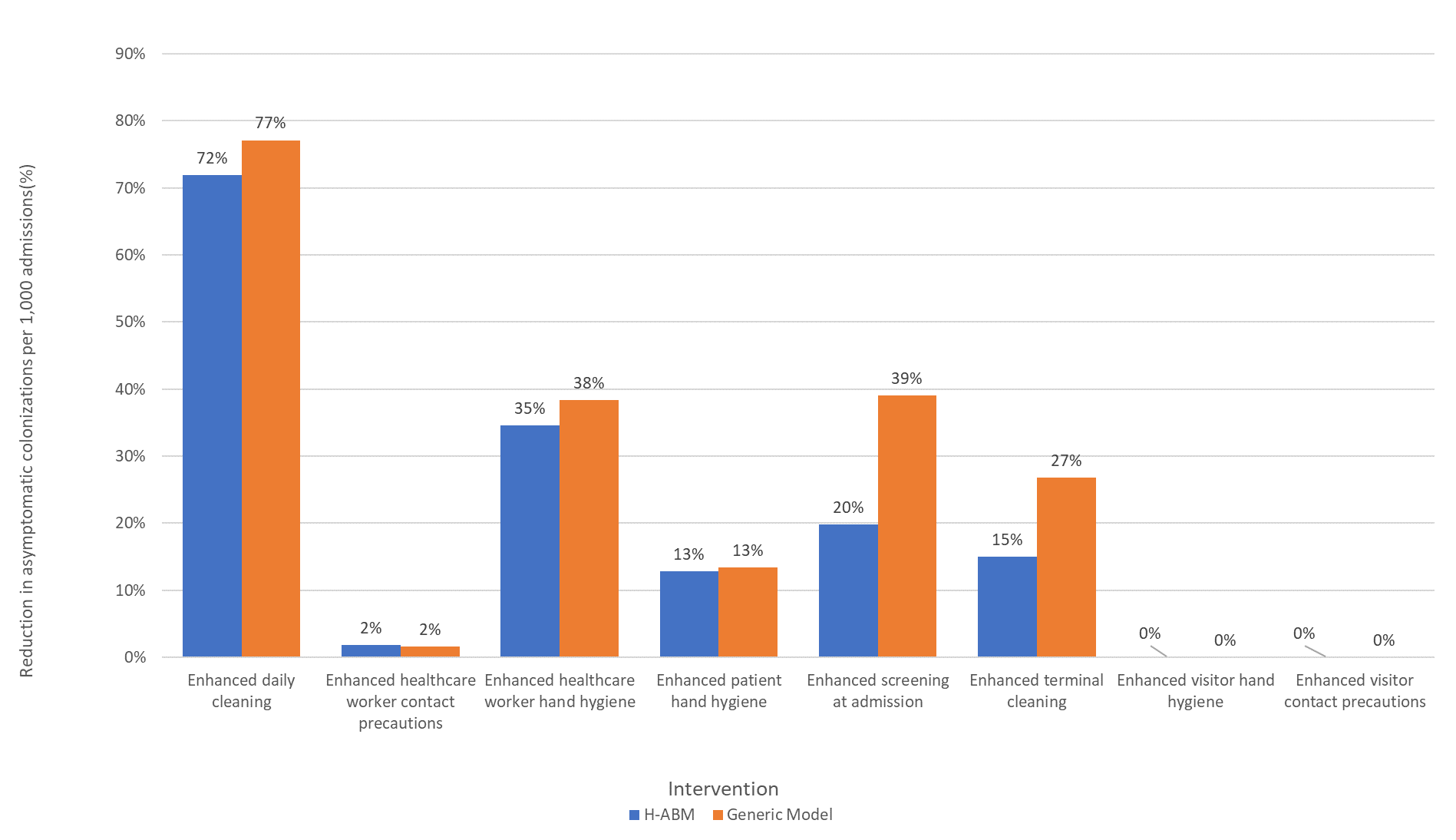


(b)


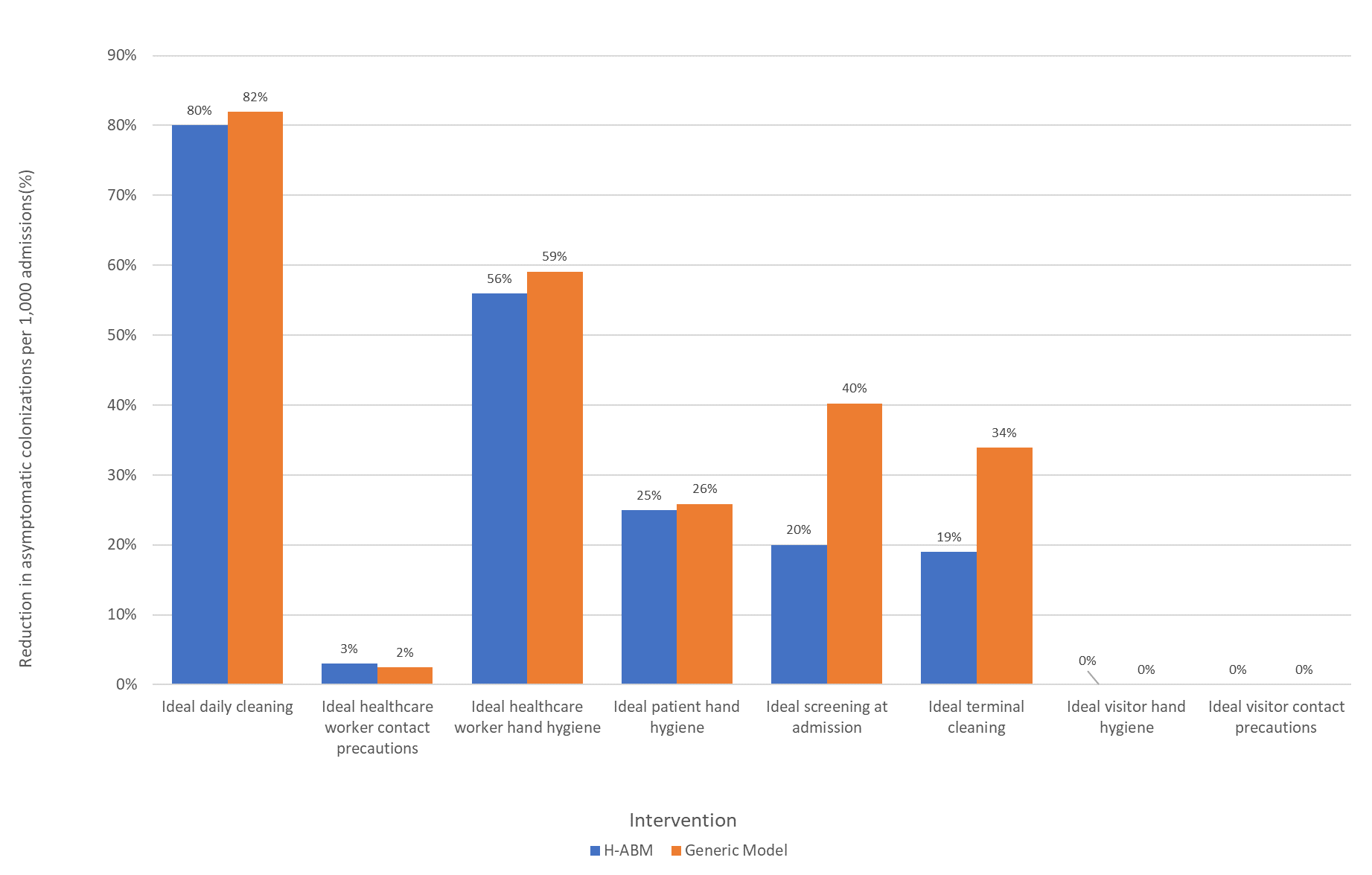


**Supplement section G. Sensitivity Analysis**

*Supplement Table G1: Rate of HA-CDI per 10,000 patient days by transition probability matrix vs historical rate of HA-CDI per 10,000 patient days from target hospital*

| Year | Transition matrix number | | | | | | | | | | Actual CDI Rate |
| --- | --- | --- | --- | --- | --- | --- | --- | --- | --- | --- | --- |
|  | 1 | 2 | 3 | 4 | 5 | 6 | 7 (Baseline) | 8 | 9 | 10 |  |
| 2013 | 16.16 | 11.76 | 16.91 | 14.10 | 17.02 | 12.40 | 14.46 | 10.34 | 14.78 | 15.97 | 12.14 |
| 2014 | 16.57 | 12.06 | 17.27 | 14.48 | 17.38 | 12.70 | 14.80 | 10.56 | 15.14 | 16.39 | 13.70 |
| 2015 | 15.00 | 10.97 | 15.77 | 13.15 | 16.11 | 11.63 | 13.47 | 9.71 | 13.84 | 14.81 | 14.01 |
| 2016 | 7.87 | 5.96 | 8.58 | 7.00 | 9.03 | 6.42 | 7.23 | 5.47 | 7.60 | 7.80 | 7.58 |
| 2017 | 6.29 | 4.88 | 6.84 | 5.65 | 7.22 | 5.20 | 5.83 | 4.55 | 6.16 | 6.26 | 5.78 |
| 2018 | 6.11 | 4.74 | 6.59 | 5.53 | 6.98 | 5.07 | 5.68 | 4.43 | 5.98 | 6.09 | 6.43 |

*Supplement Table G2: Rate of HA-CDI per 10,000 patient days under varying CDI at admission parameters*

| Year | Double Colonized at admission | Double Infected at admission | Double infected and colonized at admission |
| --- | --- | --- | --- |
| 2013 | 18.14 | 16.33 | 18.08 |
| 2014 | 18.45 | 16.76 | 18.40 |
| 2015 | 17.07 | 15.25 | 17.01 |
| 2016 | 9.65 | 7.96 | 9.71 |
| 2017 | 8.03 | 6.39 | 8.10 |
| 2018 | 7.80 | 6.20 | 7.83 |

**References**

1. Codella J, Safdar N, Heffernan R, Alagoz O. An agent-based simulation model for Clostridium difficile infection control. Med Decis Making. 2015 Feb;35(2):211–29.

2. Barker AK, Alagoz O, Safdar N. Interventions to Reduce the Incidence of Hospital-Onset Clostridium difficile Infection: An Agent-Based Modeling Approach to Evaluate Clinical Effectiveness in Adult Acute Care Hospitals. Clin Infect Dis. 2018 Apr 3;66(8):1192–203.

3. Wilensky U. Netlogo [Internet]. Center for Connected Learning and Computer-Based Modeling, Northwestern University. Evanston, IL.; 1999. Available from: http://ccl.northwestern.edu/netlogo/

4. Scaria E, Barker AK, Alagoz O, Safdar N. Association of Visitor Contact Precautions With Estimated Hospital-Onset Clostridioides difficile Infection Rates in Acute Care Hospitals. JAMA Netw Open. 2021 Feb 26;4(2):e210361–e210361.

5. Koo HL, Van JN, Zhao M, Ye X, Revell PA, Jiang ZD, et al. Real-Time Polymerase Chain Reaction Detection of Asymptomatic Clostridium difficile Colonization and Rising C. difficile–Associated Disease Rates. Infect Control Hosp Epidemiol. 2014;35(6):667–73.

6. Alasmari F, Seiler SM, Hink T, Burnham CAD, Dubberke ER. Prevalence and risk factors for asymptomatic clostridium difficile carriage. Clin Infect Dis. 2014;59(2):216–22.

7. Leekha S, Aronhalt KC, Sloan LM, Patel R, Orenstein R. Asymptomatic Clostridium difficile colonization in a tertiary care hospital: Admission prevalence and risk factors. Am J Infect Control. 2013 May;41(5):390–3.

8. Loo VG, Bourgault AM, Poirier L, Lamothe F, Michaud S, Turgeon N, et al. Host and pathogen factors for Clostridium difficile infection and colonization. N Engl J Med. 2011 Nov 3;365(18):1693–703.

9. Eyre DW, Griffiths D, Vaughan A, Golubchik T, Acharya M, O’Connor L, et al. Asymptomatic Clostridium difficile Colonisation and Onward Transmission. PLOS ONE. 2013 Nov 12;8(11):e78445.

10. Longtin Y, Paquet-Bolduc B, Gilca R, Garenc C, Fortin E, Longtin J, et al. Effect of Detecting and Isolating Clostridium difficile Carriers at Hospital Admission on the Incidence of C difficile Infections: A Quasi-Experimental Controlled Study. JAMA Intern Med. 2016 Jun 1;176(6):796–804.

11. Nissle K, Kopf D, Rösler A. Asymptomatic and yet C. difficile-toxin positive? Prevalence and risk factors of carriers of toxigenic Clostridium difficile among geriatric in-patients. BMC Geriatr. 2016;16:185.

12. Kagan S, Wiener-Well Y, Ben-Chetrit E, Kashat L, Aouizerats J, Bdolah-Abram T, et al. The risk for Clostridium difficile colitis during hospitalization in asymptomatic carriers. J Hosp Infect. 2017 Apr 1;95(4):442–3.

13. Gupta S, Mehta V, Herring T, et al. A Large Prospective North American Epidemiologic Study of Hospital-Associated Clostridium difficile Colonization & Infection. Int Clostridium Difficile Symp Bled Slov. 2012 Sep;Abstract 020.

14. Hung YP, Lin HJ, Wu TC, Liu HC, Lee JC, Lee CI, et al. Risk Factors of Fecal Toxigenic or Non-Toxigenic Clostridium difficile Colonization: Impact of Toll-Like Receptor Polymorphisms and Prior Antibiotic Exposure. PLOS ONE. 2013 Jul 25;8(7):e69577.

15. Dubberke ER, Burnham CAD. Diagnosis of Clostridium difficile Infection: Treat the Patient, Not the Test. JAMA Intern Med. 2015 Nov 1;175(11):1801–2.

16. Lucado J, Gould C, Elixhauser A. Clostridium Difficile Infections (CDI) in Hospital Stays, 2009: Statistical Brief #124. In: Healthcare Cost and Utilization Project (HCUP) Statistical Briefs [Internet]. Rockville (MD): Agency for Healthcare Research and Quality (US); 2012 [cited 2017 Feb 19]. Available from: http://www.ncbi.nlm.nih.gov/books/NBK92613/

17. Evans ME, Simbartl LA, Kralovic SM, Jain R, Roselle GA. Clostridium difficile Infections in Veterans Health Administration Acute Care Facilities. Infect Control Hosp Epidemiol. 2014;35(8):1037–42.

18. Cohen B, Hyman S, Rosenberg L, Larson E. Frequency of Patient Contact with Health Care Personnel and Visitors: Implications for Infection Prevention. Jt Comm J Qual Patient Saf. 2012 Dec;38(12):560–5.

19. California Code of Regulations. Nurse service staff 2003. 22 C.C.R., Sec. 70217 [Internet]. 2003. Available from: https://govt.westlaw.com/calregs/Document/I8612C410941F11E29091E6B951DDF6CE?viewType=FullText&originationContext=documenttoc&transitionType=CategoryPageItem&contextData=(sc.Default&bhcp=1

20. Spetz J, Donaldson N, Aydin C, Brown DS. How Many Nurses per Patient? Measurements of Nurse Staffing in Health Services Research. Health Serv Res. 2008 Oct;43(5 Pt 1):1674–92.

21. Aiken LH, Clarke SP, Sloane DM, Sochalski J, Silber JH. Hospital Nurse Staffing and Patient Mortality, Nurse Burnout, and Job Dissatisfaction. JAMA. 2002 Oct 23;288(16):1987–93.

22. American Hospital Association. AHA Hospital Statistics. 2016.

23. Evans HL, Shaffer MM, Hughes MG, Smith RL, Chong TW, Raymond DP, et al. Contact isolation in surgical patients: A barrier to care? Surgery. 2003 Aug;134(2):180–8.

24. Barker AK, Codella J, Ewers T, Dundon A, Alagoz O, Safdar N. Changes to physician and nurse time burdens when caring for patients under contact precautions. Am J Infect Control. 2017/03/13 ed. 2017 May 1;45(5):542–3.

25. Morgan DJ, Pineles L, Shardell M, Graham MM, Mohammadi S, Forrest GN, et al. The effect of contact precautions on healthcare worker activity in acute care hospitals. Infect Control Hosp Epidemiol. 2013 Jan;34(1):69–73.

26. Rubin MA, Jones M, Leecaster M, Khader K, Ray W, et al. (2013) A Simulation-Based Assessment of Strategies to Control Clostridium Difficile Transmission and Infection. PLOS ONE 8(11): e80671. https://doi.org/10.1371/journal.pone.0080671

27. McArdle FI, Lee RJ, Gibb AP, Walsh TS. How much time is needed for hand hygiene in intensive care? A prospective trained observer study of rates of contact between healthcare workers and intensive care patients. J Hosp Infect. 2006 Mar;62(3):304–10.

28. Cross KW, Turner RD. Factors affecting the visiting pattern of geriatric patients in a rural area. Br J Prev Soc Med. 1974 May;28(2):133–9.

29. Eriksson T, Bergbom I. Visits to intensive care unit patients--frequency, duration and impact on outcome. Nurs Crit Care. 2007 Feb;12(1):20–6.

30. Gonzalez CE, Carroll DL, Elliott JS, Fitzgerald PA, Vallent HJ. Visiting preferences of patients in the intensive care unit and in a complex care medical unit. Am J Crit Care. 2004 May;13(3):194–8.

31. Fumagalli S, Boncinelli L, Lo Nostro A, Valoti P, Baldereschi G, Di Bari M, et al. Reduced cardiocirculatory complications with unrestrictive visiting policy in an intensive care unit: results from a pilot, randomized trial. Circulation. 2006 Feb 21;113(7):946–52.

32. Rock C, Pana Z, Leekha S, Trexler P, Andonian J, Gadala A, et al. National Healthcare Safety Network laboratory-identified Clostridium difficile event reporting: A need for diagnostic stewardship. Am J Infect Control. 2018 Apr 1;46(4):456–8.

33. Dunn AN, Radakovich N, Ancker JS, Donskey CJ, Deshpande A. The Impact of Clinical Decision Support Alerts on Clostridioides difficile Testing: A Systematic Review. Clin Infect Dis. 2021 Mar 15;72(6):987–94.

34. Centers for Medicare & Medicaid Services. HAC-Reduction-Program [Internet]. 2019 [cited 2019 Jul 29]. Available from: https://www.cms.gov/Medicare/Medicare-Fee-for-Service-Payment/AcuteInpatientPPS/HAC-Reduction-Program.html
