# Supplementary material for: Validating agent-based simulation model of hospital-associated Clostridioides difficile infection using primary hospital data
Source: PLoS One. 2023 Apr 21;18(4):e0284611. doi: 10.1371/journal.pone.0284611 (PMC10120937; doi:10.1371/journal.pone.0284611)
Supplement: Attachment — Submitted filename: REVIEWER REPORT 20_12_22.docx [file pone.0284611.s002.docx]

**Title: VALIDATING AGENT BASED SIMULATION OF HOSPITAL ACQUIRED CLOSTRIDIODES DIFFICILE INFECTION USING PRIMARY HOSPITAL DATA**

The study was aimed at demonstrating the use of an alternative approach to validating an existing generic agent based model of Clostridiodes difficile infection using hospital specific conditions, also to propose a new parameter for validating the socio-environmental network structure of agent based models (ABM).

The authors adapted a generic ABM into a targeted 426 bed hospital setting (H-ABM) which represented Clostridium difficile infection rate within the hospital. They used Infection Prevention and Control behaviour of healthcare workers, hospital layout and other parameters to predict Clostridiodes Difficile infection (CDI) rate which was compared to the actual observed infection rate between 2013-2018.They used colonization pressure as an indicator to validate the social economic network in the model. The authors also tried to compare and rank the performance of individual IPC interventions in the generic ABM and H-ABM.

The H-ABM showed similar trends of CDI within 2013-2018 to that actually observed in the hospital. The model also showed a 46% drop in CDI following higher compliance to IPC measures. Increase CDI in the socio-environmental network resulted to an increase in the risk of Clostridiodes difficile colonization.

My overall impression is that this is a novel study where the authors tried to validate an already established model for infectious disease. This is critical to building trust in the output of these models given their utility in decision making by infection prevention and control experts within health facilities and in outbreak situations. The research attempts to circumvent the issues of extensive data requirements of the generic ABM while validating it. . The abstract and introduction were well written. There is however the need to elaborate further on the burden of Clostridiodes difficile in terms of its prevalence as a HAI and the significance of its morbidity and mortality associated with in order to put the relevance of this study in context.

The lack of data on patients and visitors IPC behaviour limits the generalization of the findings of this study within the hospital setting. Table 1 is confusing, the authors need to provide an explanation for the two percentages within each cells. The discussion provides an explanation for the findings but the research team have not sufficiently discussed their findings in the context of similar published works. The conclusions are in line with the objectives of the study and show the use of the H-ABM as an external validation model for the generic ABM and the significant utility derived from conducting a hospital specific ABM.

I personally think this is a well written manuscript, the findings are evidence that should be carefully considered by epidemiologists and IPC experts. I recommend that the manuscript be published after the raised queries within this review have been addressed.
