# Supplementary material for: Validating agent-based simulation model of hospital-associated Clostridioides difficile infection using primary hospital data
Source: PLoS One. 2023 Apr 21;18(4):e0284611. doi: 10.1371/journal.pone.0284611 (PMC10120937; doi:10.1371/journal.pone.0284611)
Supplement: Attachment — Submitted filename: Reviewers Comment_Rahab Charles Amaza_CDI.docx [file pone.0284611.s003.docx]

**General comments**

1. This is an important study with interesting findings. It is quite commendable and will add to the body of knowledge.
2. Have a uniform in-text referencing according to the journal guideline. Sometimes, the references come after the period and other times, the references appear before the period.

**Minor corrections**

**Abstract**

The abstract should take an IMRAD structure (Introduction, Methods, Results and Discussion) and there is also no need for the headings to be spelt out into the various components, it should just assume a flow.

**Introduction**

1. Line 7- ISPOR-SMDM should be spelt out in full for future references
2. Line 16 to 31– Should be referenced
3. Line 35- *Clostridium difficile* infection should be term CDI not (*C. difficile*; CDI)
4. Line 37- Change “adapt’’ to adapted
5. Line 43- Change ‘’compare’’ to compared

**Methods**

Change ‘’CDI Background’’ to Background or introduction

**Overview of the existing agent-based simulation model developed for a generic hospital setting**

1. Line 7 to 22 – It is too lengthy, should be rephrased and properly referenced

**Modeling infection control interventions**

1. Line 5- Change ‘’define’’ to defined

**Validation of socio-environmental network structure via colonization pressure**

1. Line 5 to 13- Should be referenced
2. Line 12- Grammatical error should be addressed

**Discussion**

In a s much as there was detailed discussion which included the conclusion, the conclusion should be separated from it to stand on its own.

Line 22- Paraphrasing and referencing will be preferred rather than using “the existing literature’’ and referencing the literature, kindly effect changes.

**List of Abbreviations**

Several acronyms were used in your manuscript hence a list of abbreviations used should be made available.
