# Supplementary material for: Validating agent-based simulation model of hospital-associated Clostridioides difficile infection using primary hospital data
Source: PLoS One. 2023 Apr 21;18(4):e0284611. doi: 10.1371/journal.pone.0284611 (PMC10120937; doi:10.1371/journal.pone.0284611)
Supplement: Attachment — Submitted filename: Re-review of manuscript.docx [file pone.0284611.s005.docx]

**Title: VALIDATING AGENT BASED SIMULATION OF HOSPITAL ACQUIRED CLOSTRIDIODES DIFFICILE INFECTION USING PRIMARY HOSPITAL DATA**

Following the review of the updated manuscript, I think that the authors have to a very great extent addressed the queries raised. The burden of CDI has been expressed in terms of its morbidity and mortality although the prevalence of this issue was not stated. The authors have also included as one of its limitation, the lack of data on patient and visitors IPC behaviour. Table 1 has been properly formatted to allow for easy understanding and interpretation of the numbers in the cells. The discussion section has also been done with citation of more relevant literature.

I am satisfied with the current update on the manuscript as the authors have taken the time to address point by point all issues raised. I therefore recommend that the manuscript be published.
