# Supplementary material for: Validating agent-based simulation model of hospital-associated Clostridioides difficile infection using primary hospital data
Source: PLoS One. 2023 Apr 21;18(4):e0284611. doi: 10.1371/journal.pone.0284611 (PMC10120937; doi:10.1371/journal.pone.0284611)
Supplement: Attachment — Submitted filename: General Comments_CDI Manuscript.docx [file pone.0284611.s006.docx]

**General Comments**

The manuscript is systematic and well put together, previous recommendations for corrections have been effected. However, in your discussion, it is always important to clearly state the health/public health implication of the work and how it has added to the body of knowledge.
